# A Novel Approach for Dynamic (4d) Multi-View Stereo System Camera Network Design

**DOI:** 10.3390/s22041576

**Published:** 2022-02-17

**Authors:** Piotr Osiński, Jakub Markiewicz, Jarosław Nowisz, Michał Remiszewski, Albert Rasiński, Robert Sitnik

**Affiliations:** 1 STARS Impresariat Filmowy SA, 8 Józefa Str., 31-056 Cracow, Poland; jakub.markiewicz@pw.edu.pl (J.M.); j.nowisz@mnemosis.pl (J.N.); m.remiszewski@mnemosis.pl (M.R.); a.rasinski@mnemosis.pl (A.R.); robert.sitnik@pw.edu.pl (R.S.); 2 Institute of Micromechanics and Photonics, Warsaw University of Technology, 8 Sw. Andrzeja Boboli Str., 02-525 Warsaw, Poland; 3 Faculty of Geodesy and Cartography, Warsaw University of Technology, Pl. Politechniki 1, 00-661 Warsaw, Poland

**Keywords:** Multi-View Stereo, dense point cloud, view planning, image network, OpenMVS

## Abstract

Image network design is a critical factor in image-based 3D shape reconstruction and data processing (especially in the application of combined SfM/MVS methods). This paper aims to present a new approach to designing and planning multi-view imaging networks for dynamic 3D scene reconstruction without preliminary information about object geometry or location. The only constraints are the size of defined measurement volume, the required resolution, and the accuracy of geometric reconstruction. The proposed automatic camera network design method is based on the Monte Carlo algorithm and a set of prediction functions (considering accuracy, density, and completeness of shape reconstruction). This is used to determine the camera positions and orientations and makes it possible to achieve the required completeness of shape, accuracy, and resolution of the final 3D reconstruction. To assess the accuracy and efficiency of the proposed method, tests were carried out on synthetic and real data. For a set of 20 virtual images of rendered spheres, completeness of shape reconstruction was up by 92.3% while maintaining accuracy and resolution at the user-specified level. In the case of the real data, the differences between predictions and evaluations for average density were in the range between 33.8% to 45.0%.

## 1. Introduction

Currently, two groups of methods for 3D shape reconstructions are commonly used, namely, passive (image-based) and active (range-based) methods [1,2,3,4,5,6,7,8]. Along with LiDARs, Time of Flight (ToF) cameras, and structured light (SL) systems (types of the range-based methods), one of the most popular types of sensors used to generate an object’s 3D shape is stereovision, or multistereo-vision, a system that is based on cameras and the mathematical relationships between corresponding features detected on images. The algorithms used for 3D shape reconstruction from multiple images are usually based on a combination of Structure-from-Motion (SfM) and Multi-View Stereo (MVS) methods [9,10]. These techniques are commonly used for the realisation of video game assets, virtual reality, augmented reality and virtual tours [11,12], cultural heritage preservation and protection [7,13,14], computer graphics, animation [13,15,16,17,18], and more.

To reconstruct 3D shapes, it is necessary to correctly arrange the cameras around the test object. At present, there is no standard procedure for image-based 3D reconstruction for shape determination. However, it is possible to determine the minimum needs for a network of images in order to guarantee maximum coverage and accuracy using the minimum number of sensor positions. To fulfil these requirements, the following set of parameters defining the spatial camera distribution should be considered: (1) The base-to-depth (B/D) ratio, also called base-to-height (B/H) ratio, where the network accuracy increases when this ratio increases when using convergent images rather than images with parallel optical axes [19]. (2) The number of acquired images, where the accuracy improves significantly with the number of images in which a point appears. It should be stressed that measuring points on a number of images greater than four does not significantly affect accuracy [20]. (3) The number of measured points in each image, where the accuracy increases with the number of measured points per image. However, the increase is insignificant if the geometric configuration is strong, and the measured points are well defined (such as targets) and well distributed in the image. In addition, this also applies for the control point utilised [20]. (4) The image resolution, where, for natural features, the accuracy improves significantly with increasing image resolution, while this improvement is less significant for well-defined largely resolved targets [21].

This article proposes a novel approach for designing and planning a multi-view imaging network for dynamic 3D scene reconstruction in the absence of initial information about the object’s geometry or specific location, which is one of the most critical components of the 3D shape reconstruction pipeline using the SfM/MVS approach. The proposed method assumes that the only constraint is that the moving object is placed inside a defined measurement volume that is much larger than the object itself, and a single sensor’s field of view (FOV).

Compared to other methods that assume that the approximate 3D shape and position are static and do not change during image acquisition, in the proposed method, the object may undergo any transformation, as long as it remains inside the specified volume during surveying. This means that the proposed algorithm is suited for static scenes, in which the object can be placed in any position within the measurement volume, as well as for dynamic scenes, where the object is moving inside the volume. Moreover, our proposed algorithm allows operation not only under any transformation, but also with different sensor properties, namely focal length, resolution, and pixel size. Both sensor transformations and properties are used to obtain full coverage in the reconstruction, as well as the required accuracy and density, based on the number of optical sensors. The user must specify the measurement volume, the sensors used, and their properties. Next, the volume is discretised using a regular point grid. The Monte Carlo algorithm is used; thus, the sensor setup is randomly drawn, and the predictions of the coverage, density, and accuracy of the reconstruction are calculated for each point in the specified directions. The main contributions of this paper are the proposed prediction functions for coverage, density, and accuracy, computed in the angular domain. A quasi-optimal sensor setup that fulfils the given requirements is calculated on the basis of those predictions. To the best of the authors’ knowledge, currently, there are no solutions to the given problem that enable the design of a image network for the purposes of SfM or MVS reconstruction based on measurement volume alone. Moreover, there is no approach that enables the optimised prediction of measurement coverage, density and accuracy using calculated reconstructions (only theoretical formulas are used in scientific papers).

This paper is divided into six main sections: Section 2 surveys previous work related to image network design for 3D shape reconstruction. Section 3 contains a description of the test scenarios and the approach used for data analysis. In Section 4, the results of the performed experiments are summarised, and these are later discussed in Section 5. In the conclusion, Section 6, future work is proposed, and the possibilities and limitations of the proposed image network design are summarised.

## 2. Related Work

Image network design is one of the most critical steps in image-based 3D reconstruction and data processing (especially in the application of combined SfM/MVS methods), impacting the final accuracy and, most importantly, the completeness of the final 3D models. Due to this, multiple approaches have been developed for different applications and sensor types. Still, most of them assume prior knowledge about an object or a partial reconstruction from online processed data [16]. Conversely, most image network design approaches rely on the general rules proposed by Fraser [19], which determine the selection of objects and base-to-height (B/H) ratio, the improvement of accuracy with increasing number of views, what focal lengths are to be employed, or how many exposures to select. In general, image network design algorithms for image-based 3D reconstruction can be divided into those that address static and dynamic objects.

The problems of image network design for static objects have been well described, and such techniques are commonly used for the digitalisation of cultural heritage objects [17,18] and close-range [18,19,22,23] or aerial [24,25,26] applications. These applications are usually based on rough reconstructions using the next-best-view algorithm [27,28]. This method plans the optimal number of views necessary to achieve the required accuracy while maintaining maximum object coverage. An image network design algorithm for high-density point reconstruction (above 1000 points/mm^2^) was described in Karaszewski et al. [17]. The authors proposed an algorithm based on clustering surfaces by normal vectors from a rough reconstruction. The proposed approach used an SL pair mounted on a robotic arm for the initial point cloud generation and final 3D shape reconstruction. After the use of the initial 3D shape, a normal vector histogram was calculated to cluster the surfaces, determining the views for each cluster (structured light system position). The best path is calculated in the next step, and the scan is processed. Finally, holes and low-density areas on the reconstruction are detected and rescanned. A similar approach could be used for large and complex monuments. Alsadik et al. [18] proposed a method that assumed the calculation of a rough reconstruction using a video stream, which is then decomposed into multiple subdivisions (the reconstruction of an object with multiple flat surfaces can be assumed if the object is a building). Next, the initial image network for each subdivision is generated based on the image overlap ratio. The number of views is later optimised by removing cameras that are redundant in terms of accuracy and density. Finally, a nonlinear optimiser is used to calculate the ultimate view positions. The accuracy and density are determined by estimating the minimum and maximum distances from the object and the B/H ratio [19]. Another example of using image network design for buildings with the use of an unmanned ground vehicle (UGV) was published by Hosseininaveh and Remondino [23]. Similar to the previously described algorithms, an initial 3D model is required. The proposed method relied on a candidate views grid. The candidates are limited to only two heights, because of the use of the UGV. The views are limited to those where the distance to the object lies within the camera’s depth of field (DoF). The orientations of the views are calculated with one of three proposed approaches (facing centre of the building, nearest building point, or both). Finally, visibility matrices (rough cloud points visible to each candidate view) are calculated, and on the basis of these, procedures for vantage point selection are also implemented. This approach was validated on the basis of real and synthetic data.

Another application of network design methods is to determine optimal unmanned aerial vehicle (UAV) flight plans for reconstructing non-topographic objects [22,29]. Apart from a few additional problems such as safety, stability, and flight time, in general, the approaches to view planning are similar to those in the previously described applications. Slightly different from this are algorithms for topographic measurements [25,26]. These generally consider parameters such as map scale (or ground sampling distance), camera type, and aeroplane/UAV speed. The optimal path is reduced to a 2D problem. The minimum image overlap (both forward and sideways) is set, and the height from which images are captured, the base length (offset between images), and the maximal exposure time are calculated.

The next level of complexity in image network design is represented by the reconstruction of dynamic scenes. Currently, only a few solutions to this issue have been presented. Some of these consider dynamic objects in the scene and use moving cameras. Generating the optimal formation of UAVs, and maintaining this while following a target for motion capture calculation, was presented in [30,31,32,33]. Similarly, moving cameras for the capture of dynamic scenes were described in [34], for the purposes of reconstructing surgical cavities. However, those solutions used rather trivial approaches to the problem of image network design. Another important approach to image network design for motion capture systems (mocap) was presented by Rahimian and Kearney in [35], and further extended in [36]. The goal was to design an optimal camera image network for detecting markers attached to an actor moving inside a predefined area. They proposed two approaches to the problem. The first was occlusion-based, and computed target point visibility in the presence of dynamic occlusions with cameras with “good” views. The cost function consisted of three parts: (1) the first comprised point 3D reconstruction conditions, which simply define some range in the stereo-pair axis angles for which the required accuracy is fulfilled (a 40–140-degree range, based on the criteria defined in [37]). (2) Similarly, the resolution degradation condition was defined as a given distance range to the camera in which a marker was visible to the camera. (3) Finally, dynamic occlusions were modelled using probabilistic functions, proposed by Chen and Davis [38], as a vertical plane rotating around a point for which only half of the plane is visible. The ratio of angles for which the stereovision condition is fulfilled was considered to be the dynamic occlusion cost. Moreover, the authors proposed computation optimisation for the calculation of dynamic occlusion. The second approach was distribution based, where the goal was for the target to be seen from the broadest range of viewpoints. The target point was approximated with a sphere, and uniform points were determined. Subsequently, a maximum average minimum spanning tree (MST) was calculated on the basis of the camera’s directions, and its size was treated as the cost function. This proposed method optimised one of the described cost functions, calculated for the defined target point sets using the Simulated Annealing (SA) algorithm [39,40]. The optimiser found a solution close to the global minimum by moving a single camera in each step. The cameras had defined properties and could be placed in predefined lines, planes, or areas. In [41], the authors proposed a guided generic algorithm based on crossover and mutation strategies on visibility markers vs. a camera matrix to optimise camera count. They used simple metrics as a resolution range in order to satisfy accuracy requirement, for the field of view, including obstacles in the pinhole model, and under conditions in which at least two cameras had to see the marker for it to be reconstructed. This algorithm is suited to convergence on the basis of criteria of quality (optimal camera network), calculation time, or cost (camera count).

## 3. Materials and Methods

**Figure 1:**
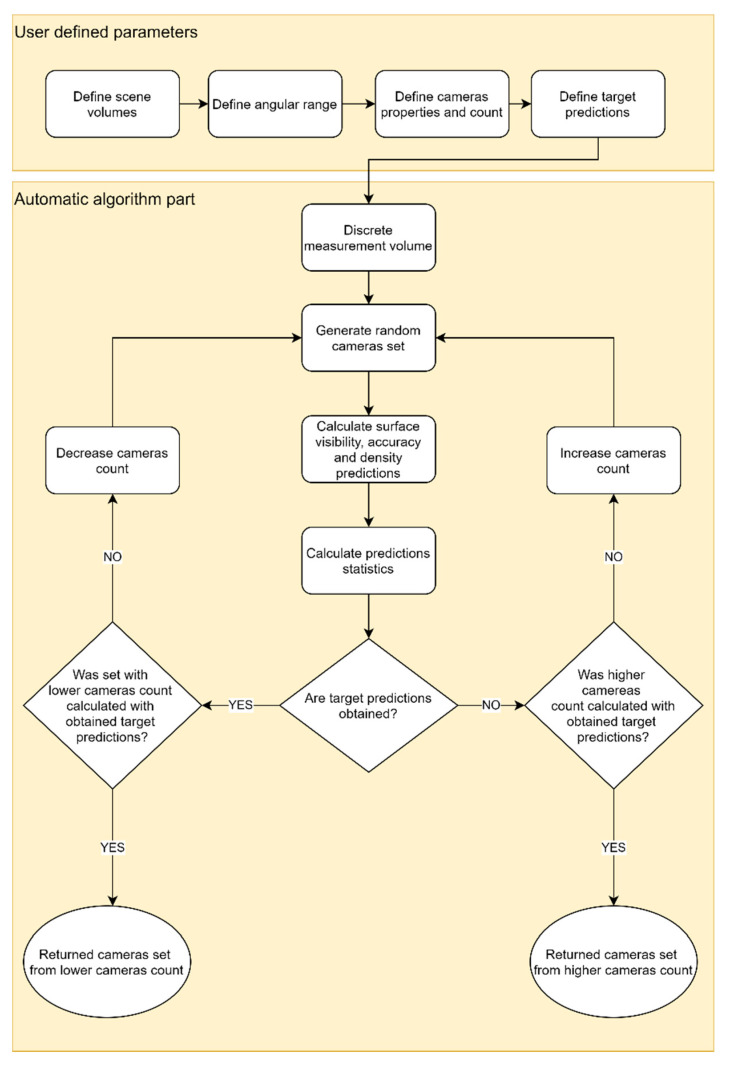
Scheme of the proposed views network planning algorithm.

The proposed methodology for an automatic camera network design algorithm uses a multi-stage approach. It is based on original software and consists of two main stages (Figure 1): (1) determine the input parameters that might be different for each scenario, which are defined manually by the user, and (2) determine the main algorithm for determining the camera position, discretising the measurement volume using a regular point grid and a Monte Carlo-based algorithm. The best camera setup is selected.

The idea of the image network design algorithm presented in this paper (according to the diagram shown in Figure 1) contains the following steps:

1. Set input parameters:
  ○ Define scene volumes:
    ■ Measurement volumes;
    ■ Permitted volumes/areas/lines for sensors positions.
  ○ Define angular range for which predictions are calculated.
  ○ Define number of available cameras and their properties.
  ○ Define target coverage, density, and accuracy for each measurement volume.
2. Automatic algorithm:
  ○ Discretise measurement volumes with points in which predictions will be calculated.
  ○ Repeat the following procedure until the stop condition is fulfilled:
    ■ Generate a random camera setup.
    ■ Calculate coverage, accuracy, and density predictions.
    ■ Calculate statistics from predictions.
    ■ Check if required predictions are achieved:
      - If yes, and lower camera count was not checked, decrease camera count and repeat generating camera setups.
      - If yes and the requirements are fulfilled for lower camera count, select as a quasi-optimal set.
      - If no and higher camera count was not checked, increase camera count and repeat generating camera setups.
      - If no and higher camera count was already calculated and the requirements were fulfilled, select the best result from a higher camera count.

Optionally, different scenarios are possible for the stop condition. For example, sometimes, it is more convenient to generate a given number of camera setups and manually choose the best one.

Defining prediction functions and setting their properties is crucial for obtaining valid predictions, and thus an optimal camera network. Hence, this section was divided into two main subsections. First, the methodology for designing the prediction functions and selecting their parameters with reference to the reconstruction of a synthetic dataset is described. The second subsection characterises the camera network design algorithm itself.

### 3.1. Prediction Functions

The main properties of the 3D Multi-View Stereo reconstruction are its coverage, accuracy, and density. Thus, these are the three factors that need to be included in the cost function for determining the optimal camera setup, and therefore, they need to be predicted. Theoretical equations that define coverage, accuracy, and density are available. However, these are only general concepts and, compared to real measurements and specific reconstruction algorithms, they need to be adapted and their parameters need to be determined. Several prediction functions were tested, and the best ones were chosen. The selection was made on the basis of quantity assessment by comparing the synthetic data with the ground truth.

#### 3.1.1. Dataset Preparation

Two types of synthetic dataset with known ground truth were prepared to design valid prediction functions. The first one consisted of multiple camera setups placed on the *XY* plane in different configurations, with the measured object positioned at several different distances on the *Z*-axis. This was used for prediction function optimisation. The second type was a validation dataset, and was an imitation of a 360-degree scenario with cameras mounted on columns around the measurement area. For the purposes of this paper, cameras with properties equivalent to a Flir Oryx 10Gige with 4k resolution, which allows the measurement of dynamic scenes at 68 frames per second, were used [42]. Lenses with a focal length of 16 mm were chosen. In this paper, the rendered images were considered to be synthetic data. The renders were generated from simulated cameras using Autodesk 3dsmax 2020 [43], and spheres were used as the objects to be rendered for the purposes of the dataset.

**Figure 2:**
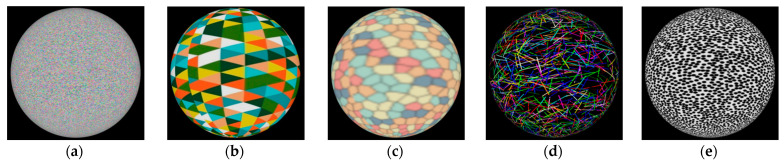
Example of the sphere with rendered texture used for final validation: (**a**) RGB noise (random colours), (**b**) colour triangles, (**c**) stained glass, (**d**) random colour line segments, and (**e**) random black points.

In the proposed view planning algorithm, several simplifications were applied: the images were devoid of geometric and radiometric distortion, the same texture was selected for all objects, spheres were used as the test objects, and a pinhole camera model was employed. Moreover, occlusions were not taken into account; therefore, each object was rendered separately. Moreover, no impact of texture was taken into account. Therefore, the texture used was selected in order to obtain the best possible reconstruction (with respect to coverage, density, and accuracy). For this purpose, five different textures (Figure 2) were tested. To analyse the influence of the texture on the quality of the 3D shape using the MVS approach, 52 synthetic camera images were used to reconstruct a sphere with a radius of 500 mm (Table 1). For all 3D reconstructions presented in this paper, OpenMVS [44] version 1.1.1 was used.

Five texture types were tested (Figure 2, Table 1), and RGB noise (random colours) was chosen with a frequency such that the Nyquist criterion [45] was fulfilled for all rendered images. Although the random black points texture resulted in a slightly more dense reconstruction, it was significantly less accurate.

Finally, Autodesk 3ds Max 2020 was used to render synthetic camera images with specific parameters and transformations. These images were used as the input for the MVS reconstruction algorithm, and further reconstructions were used to compare the prediction results. Because the authors’ algorithm does not take photometry effects into account, the lights are not included in the rendering process; rather, only a “diffuse” map is used in 3ds Max; this is referred to as “diffuse albedo”—Arbitrary Output Variables (AOV) [46].

**Figure 3:**
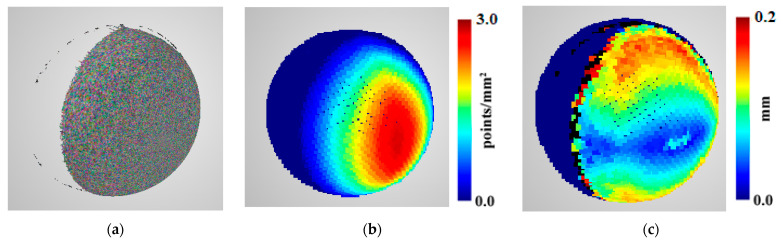
Example (**a**) sphere reconstruction from a two-camera scenario; (**b**) the results of density computation (points/mm^2^); (**c**) accuracy evaluation (mm).

To assess our predictions and compare them to reconstructions obtained using OpenMVS, evaluations were calculated. The evaluations were sparse, quasi-equidistant point clouds covering the ground truth object’s surface, for which local reconstruction coverage, density, and accuracy were calculated. For each sphere, points at a distance of 30 mm were used for evaluations, resulting in 4444 evaluation points. Furthermore, they were used to assess and choose the best prediction functions. An example sphere reconstruction and its assessment is presented in Figure 3.

**Figure 4:**
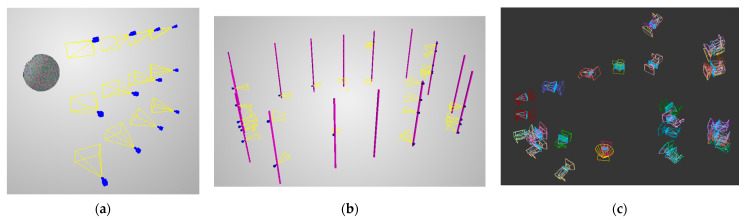
Example of a (**a**) 12-camera setup with the reconstructed sphere; (**b**) 360-degree scene with camera distribution view with columns; (**c**) print screen from 3dsmax of the same 360-degree scene.

The first dataset consisted of setups with 2, 3, 4, 6, 9, or 12 cameras, respectively, placed on the *XY* plane at distances of 1 m in different configurations, with the sphere positioned at four distances on the *Z*-axis. In the two-, three-, and four-camera setups, the cameras were arranged in a single horizontal row; the six-, nine-, and twelve-camera setups were formed in 2 × 3, 3 × 3, and 4 × 3 camera arrays, respectively. For each scenario, the sphere was positioned at the following distances from the camera array plane: 2, 3, 4, and 5 m, in two variants each: on the cameras’ symmetry axis and off the symmetry axis. This resulted in 48 reconstructed spheres in total. As an example, the reconstructed sphere from the 12-camera scenario are presented in Figure 4a.

The pipeline for generating 360-degree validation scenes is as follows:

- Generate random camera setups.
- Randomly place cameras on permitted columns.
- For each, select a random point within the measurement volume to look at.
- Add synthetic objects to the scene.
- Render images from all cameras.

For the purposes of this paper, there were 10,000 random 360-degree scenes generated. Each of them consisted of a cuboid measurement volume of 2.6 × 4.0 × 2.0 m with columns around it, on which 20 cameras were mounted. The random camera positions were processed by drawing a single column for each camera, as well as the camera’s height on the column. Next, a random point from measurement volume was selected for the camera to look at. All cameras were rotated horizontally. The full description of the generation of random camera setups is described in Section 3.2.4, and an example camera setup is presented in Figure 4b,c.

#### 3.1.2. Prediction Parameter Optimisation

The selection of the prediction function was based on a comparison of the evaluations from the dataset described in Section 3.1.1. Because some predictions had unknown parameters specific to the given reconstruction algorithm, these were optimised to obtain the best correlation with the evaluations. The least-square Levenberg–Marquard optimiser was used to determine unknown parameters [47]. In each optimiser iteration, the predictions were compared to evaluations from the whole dataset. A given prediction function was compared to another, and was tested after the unknown parameters had been optimised. The proposed prediction functions were chosen and optimised specifically for use in the OpenMVS reconstruction algorithm. Different reconstruction algorithms may result in further evaluations of values and distributions, and therefore, the proposed prediction functions may not be valid for them.

Because accuracy was calculated as the degree of difference between the reconstruction and the ground truth, the accuracy was stated as infinity for points at which no reconstruction was possible because of there being no suitable cameras. Such values are hard to compare and use for optimisation. For this reason, the errors were normalised. The Sigmoid function (Equation (1)) [48] was used for optimisation, as it normalises the whole (−inf, inf) range to (−1, 1). Sigmoid normalisation was used for both accuracy and density.

(1) $$y=\frac{1}{1+e^{-x}}=\frac{e^{x}}{e^{x}+1}$$

#### 3.1.3. Reconstruction Coverage

In MVS algorithms, the reconstruction pipeline is as follows: calculation of depth maps for camera pair, depth map refinement, and finally, depth map merging [49]. Therefore, before prediction functions can be assessed, it has to be determined which camera pairs are being used for a given point with a given normal vector reconstruction. Again, the optimiser was used to determine these conditions and parameters. The cost function was designed as a negative XOR function for binary-considered evaluations and predictions. The evaluation was 0 if, in the local neighbourhood, there were no reconstructed points, and 1 otherwise. Similarly, the prediction was either 1 or 0 based on the reconstruction’s coverage prediction function.

For a particular camera pair to be useful for reconstruction, some conditions need to be fulfilled. It is assumed that stereo image pairs should have a similar viewing direction and magnification, and a suitable baseline. After condition tests, it was determined that the best results were obtained when using the following conditions for the determination of valid cameras:

- The angle between optical camera axes:

Eligible stereo pairs of cameras are chosen, similar to the approach presented in [50]. First, it is assumed that for each *i*-th camera, *n* images are the potential images used for camera stereo pair determination. For all image pairs, the angle between the principal view direction of caIeras *i* and *j* is computed: $\theta_{ij}=1,\;\ldots ,\;n$. Then, the pairs of images with $\theta_{ij}<5{}^{\circ}$ or $\theta_{ij}>60{}^{\circ}$ are removed.

- The B/H ratio:

The base–height ratio generally impacts the accuracy of the depth of point calculation. In the proposed approach, the base–height ratio condition is defined using the following steps: for each pair of images, the distance between the camera centres is computed: $d_{ij}=1,\ldots ,\;n.$ Then, the median ($\overset{=}{d}$) of $d_{ij}$ is determined, and pairs of images with $d_{ij}>2\bar{d}$ or $d_{ij}<0.05\bar{d}$ are removed.

- The magnification difference between the cameras at a given point:

The patch scales on two cameras need to be similar in order to be able to match them between two images. Thus, magnification, represented in pixels, needs to be compared for two cameras and its ratio determined. For a given point, magnifications for both cameras are determined based on Equation (2), assuming that the following parameters are known: distance between camera and object, pixel size, and focal length.

(2) $$m=\frac{d}{f}\times p$$

where *m* is magnification, d is the distance to the object in millimetres, *f* is the focal length in millimetres, and *p* is pixel pitch.

According to the OpenMVS default parameters, this ratio needs to be in the range (0.2, 2.4) for the cameras to be able to form active camera pairs.

After determining the algorithm for selecting active camera pairs, the reconstruction coverage needs to be determined. In OpenMVS, the minimum number of camera pairs is not constant. A single pair is enough for the reconstruction of 3D coordinate points, where only two cameras are used to observe a given point from a given direction. However, if more cameras are observing the point, at least two pairs are needed for to perform a valid reconstruction.

Finally, the maximum angle between the surface normal vector and each camera was evaluated for the reconstruction of the object. It was determined that even surfaces with normal vectors rotated by 87 degrees against the observation direction of the camera could be properly reconstructed.

#### 3.1.4. Accuracy Prediction Function

In a normal case of stereophotogrammetry, the distance to the measured surface and its error are the result of disparity error. The best accuracy prediction function might approximate the following relations:

(3) $$Z=\frac{Bf}{dp}$$

(4) $$\sigma_{Z}=\frac{pZ^{2}}{Bf}\sigma_{d}$$

where *B* is the base of the stereo pair, *f* is the focal length, *d* is the disparity, and *p* is the pixel pitch.

Equation (4) shows that the accuracy of a point determination from a stereo pair of normal images depends on two components: *f*/*Z*, which describes the scale of the image, and *B*/*Z*, which represents the intersection angle.

Small intersection angles and image scales positively affect the ability to achieve a high degree of completeness (because of the image similarity and the good matching performance); conversely, they have a negative effect on depth precision due to the poor geometric conditions. The use of large intersection angles and large image scales provides better depth precision, but results in lower image similarity and point density.

As stated previously, the ideal pinhole model is used for the proposed camera network design algorithm; thus, errors in camera calibrations are not considered when predicting accuracy.

The proposed accuracy prediction method for a given point *p* with a given normal vector *n* using *k* cameras (obtained from cameras in active camera pairs) is calculated as the distance between point *p* and error point $p_{error}$, where the error point is the intersection of error projections from all cameras. Error projections are projections from the deviated sensor point $s_{deviaded}$, which is a sensor point removed from the point *p* projection by a distance equal to the feature detection error $fd_{error}$ in the direction of the camera’s middle point. Feature detection error (Equation (5)) consists of three components: a constant pixel error component, one that considers the angle between the surface normal vector to the camera observation direction, and a component depending on the number of cameras, *k*:

(5) $$fd_{error}=pixel\_erorr\times \left(1+\sin\left(\alpha \right)+log_{2}k\right)$$

where *α* is the angle between the vector from the middle point between the cameras and a given point, and opposite from its normal vector. Pixel error is determined by the optimisation process for the dataset. It is worth mentioning that variables from Equation (4), such as base *B* and distance to point *Z*, are also included in the proposed Equation (5) for calculating the intersection of projections from both cameras.

Despite what was stated in the introduction about greater numbers of cameras resulting in better accuracy, our tests reveal that this reliance is the opposite; therefore, more cameras results in lower accuracy.

#### 3.1.5. Density Prediction Function

Another parameter taken into account when determining optimal camera network design was the choice of the density function, which was explicitly related to the ground sampling distance (GSD). The desired density can be computed from a GSD (Equations (6)–(8)).

(6) $$GSD=\frac{pH}{f}$$

(7) $$r=\frac{1}{GSD^{2}}$$

(8) $$resolution=min\left\{\frac{r_{approximation}}{r},1\right\}$$

where *H* is the distance to the object, *f* is the focal length, and *p* is the pixel pitch.

Similar to accuracy prediction, we used precise estimation to determine a specific point’s 3D coordinates, unlike in [36], where they used a resolution degradation parameter, which says that the point is not visible outside a given range from the cameras.

The proposed GSD determination for a single camera is as follows:

(9) $$GSD_{cam}=\frac{1}{p_{ob}^{2}/\cos\left(\beta \right)}$$

where $p_{ob}$ is the pixel size projected on the object, and *β* is an angle between the camera’s observation direction and the opposite surface normal vector. Pixel size is calculated from Formula (10):

(10) $$p_{ob}=\frac{d_{z}\times p}{f\times \cos\left(\gamma \right)}$$

where $d_{z}\;$is the distance from the camera to the point for which the prediction is being calculated along the camera’s Z direction, *p* is pixels pitch, *f* is the camera’s focal length, and γ is an angle between the camera’s optical axis and a given point in the observation direction.

For points observed using three or fewer cameras, density prediction is calculated as the minimum of all active cameras. Otherwise, the average density of the top five cameras is considered to determine point density. The maximum number of cameras used for calculating the average value is taken from the OpenMVS number–views parameter (the number of views used for depth map estimation), which was set to 5. Thus, the predicted density *D* was calculated using the following formula:

(11) $$D=\{\begin{array}{c} \min\left(GSD_{cam}\right),\;for\leq 3\;cameras\\ \frac{\sum_{cams}^{4}GDS_{cam}}{4},\;for\;4\;cameras\\ \frac{\sum_{cams}^{5}GDS_{cam}}{5},\;otherwise \end{array}$$

### 3.2. Camera Network Design Algorithm

The proposed camera network design approach is based on the Monte Carlo algorithm [50]. It starts with setting the input parameters, namely the scene volumes, the parameters of the available cameras, and their number. Then, as presented in Figure 1, it consists of discretising the measurement volume and randomly generating multiple camera setups, calculating their predictions, and selecting the best one.

#### 3.2.1. Defining Scene Volumes

**Figure 5:**
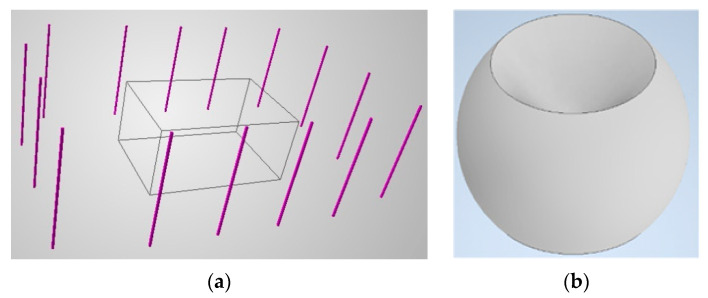
The example scene volumes: (**a**) measurement volume is presented as a black cuboid and permitted volume as segments for cameras to be mounted on as described in this paper. (**b**) Example of an angular range within which predictions are calculated (45–135 degrees in the polar direction, 0–360 degrees in azimuthal direction).

Each scene has a different geometry and restrictions, and thus, first, defining the measurement volumes and camera positions needs to be performed. These volumes may be freely defined, however, for the purposes of this paper, we define them as cuboids. We propose the definition of two types of volume (Figure 5a):

- Measurement; and
- Permitted.

The measurement volume is an area within which the measured object may move. The algorithm’s objective is for the reconstruction to fulfil a given set of surface visibility, accuracy, and density requirements. Meanwhile, permitted volume is related to camera positions. The permitted type defines the volume in which the cameras may be placed. It should be stressed that it is prohibited for the sensors to be installed inside the measurement volume. It is possible to define multiple volumes for each type, including measurement volumes with different requirements. Moreover, the following logic is proposed: a camera may be placed in a position within at least one permitted volume, but outside all measurement volumes.

Moreover, the volumes related to camera positions only can be represented with 3D surfaces or lines. The camera’s permitted volumes for this investigation were determined as 3D segments (Figure 5a).

Usually, it is not possible to mount the cameras below the scene, and sometimes the object needs to be measured from a single side only. Therefore, to avoid biased results, the normal vectors in which predictions are calculated are limited to some angular range. An example angular range is presented in Figure 5b.

#### 3.2.2. Camera Properties

Camera parameters are crucial in camera network design, because the selection of the sensor defines the quality of the images, and it is possible to obtain and define reconstruction accuracy. There needs to be a compromise between accuracy, the number of cameras, and the resolution and field of view of the individual cameras.

The choice of sensor is related to the required image quality, which is related to the radiometric signal–noise ratio for each pixel; for this reason, it is recommended to choose large sensors. Selection of the proper camera settings should always be performed to ensure sharpness within the determined sharpness range (depth of field), because this is necessary for feature point extraction (the main step in the structure-from-motion approach) and shape reconstruction using the Multi-View Stereo approach.

The choice of focal length is related to resolution, the accuracy of shape reconstruction, and field of view. Using structure-from-motion methods for image orientation makes it possible to determine the internal orientation parameters, i.e., focal length and distortion, in a self-calibration process. However, it is recommended to use fixed calibrated cameras for high-accuracy applications.

Thus, the proposed algorithm is suited for arbitrary camera resolution, sensor size, and focal length. Usually, however, this choice is narrowed to the selection of focal length only for a particular sensor.

#### 3.2.3. Discretising Measurement Volume

**Figure 6:**
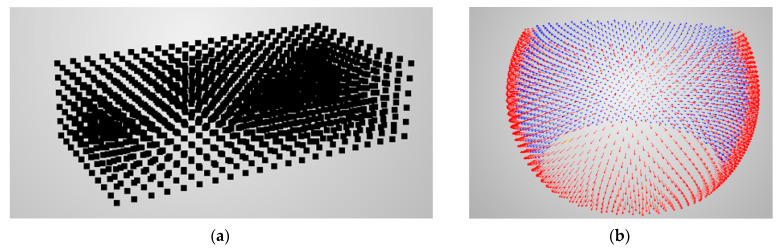
Example of (**a**) discretised measurement volume and (**b**) normal directions of single points for calculating predictions.

The proposed prediction functions (described in Section 3.1.1, Section 3.1.2, Section 3.1.3, Section 3.1.4 and Section 3.1.5) are calculated for each point in a given normal direction. Therefore, the measurement volume needs to be discretised using a grid of points with user-defined spacing (Figure 6a). Furthermore, an angular range is specified in order to calculate the predictions. Normal directions limited to the example angular range are presented in Figure 6b.

#### 3.2.4. Generating Random Camera Setup

**Figure 7:**
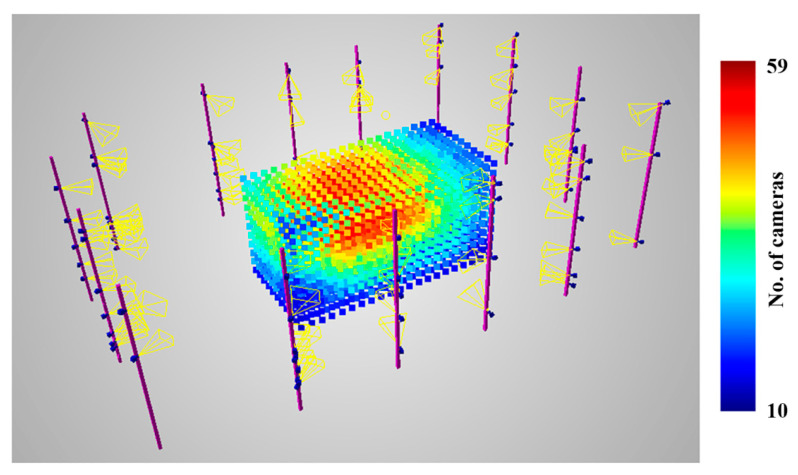
An example scene with camera setup and discretised measurement volume with the number of observing cameras presented in the colour scale.

The main idea of the proposed approach is that for each iteration of the algorithm, a random camera setup is generated. At first, a given number of cameras is generated. Possible combinations of properties can be freely designed in that the user can designate the number of cameras in each possible setup. Next, they are randomly positioned within the permitted volumes. As stated before, for the purposes of this paper, 3D segments are used to mount the cameras. Finally, camera orientations need to be selected. If truly random orientations were to be generated, many cameras would be inactive for 3D reconstruction, and most setups would be invalid. Therefore, the orientation of the cameras is defined on the basis of a randomly drawn point within a measurement volume at which the cameras a looking. Lastly, the cameras are rotated horizontally in relation to the optical axis of the cameras. An example camera setup is presented in Figure 7.

#### 3.2.5. Cost Function and Optimal Camera Setup Selection

The prediction functions were described in Section 3.1; however, this description was only related to cases in which a 3D point had a single normal vector. In order to calculate the cost, it is necessary to consider that each discrete point of the measurement volume needs to be reconstructed from all directions (within a given angular range). Therefore, instead of a single scalar value describing cost, it is necessary to generate multiple discrete directions, and calculate a vector of values for each point. The goal is to obtain a camera setup for which all measurement volume points fulfil the accuracy and density requirements from all directions, thus achieving full reconstruction coverage. In an ideal case in which the minimum requirements need to be fulfilled regardless of the number of cameras, the algorithm can be fully automatic and return a single quasi-optimal camera setup.

However, for some input parameters (i.e., an insufficient number of cameras for a measurement volume that is too large), it is impossible to fulfil all of the measurement volume requirements. Some points from some directions will not reconstructed at all. Therefore, a straightforward approach using criteria for the minimum accuracy or density to be obtained throughout the whole measurement volume in all directions is insufficient, and some statistics will need to be calculated. In such cases, the selection of the best camera setup is an ambiguous task, and we propose calculating the following statistics and allowing the operator to decide which camera setup is optimal for the given requirements:

- The ratio of fully reconstructible points;
- The ratio of reconstructible directions from all points;
- Minimal, average, median, and standard deviation of density and accuracy of reconstructible directions.

## 4. Results

The proposed image network design algorithm and parameter optimisation was implemented and tested on a synthetic dataset, as described in Section 3.1.1. The Results section is divided into three subsections. The first one contains the parameter optimisation results for single-sided scenes. The second presents the validation of one average and one quasi-optimal synthetic camera setup for a 360-degree scene, and the third presents the validation of a real 360-degree camera setup.

### 4.1. Prediction Parameter Optimisation

The prediction functions were optimised using the first dataset, which contained scenarios with different numbers of cameras placed on the XY plane and a sphere in different positions, both on and off the camera symmetry axis. This section presents evaluations, predictions, and the differences between them for a few example scenarios and the statistics for the whole dataset broken down by scenarios with different numbers of cameras.

#### 4.1.1. Reconstruction Coverage

**Figure 8:**
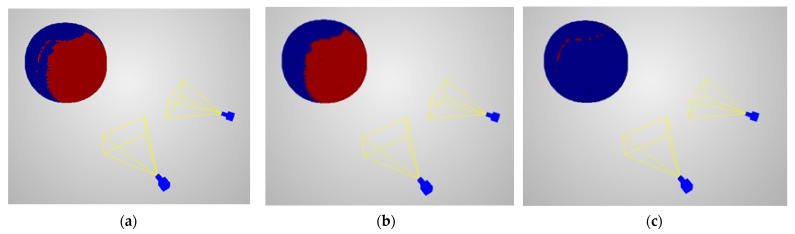
Reconstruction coverage on two camera scenarios with the sphere on the *Z*-axis at 2-m distances: (**a**) evaluations, (**b**) predictions, (**c**) differences. Red points represent areas where the sphere was reconstructed for evaluations and predictions and for differences where the evaluations differed from predictions. Blue points show otherwise.

**Figure 9:**
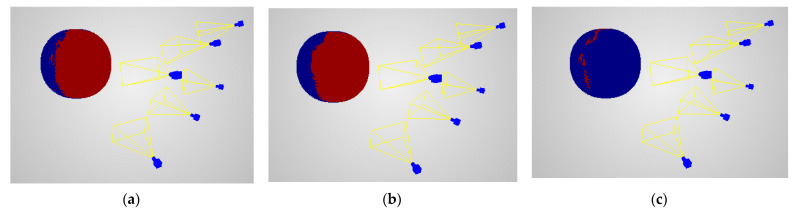
Reconstruction coverage on six-camera scenario with the sphere on the *Z*-axis at 2-m distance: (**a**) evaluations, (**b**) predictions, (**c**) differences. Red points represent areas where the sphere was reconstructed for evaluations and predictions and for differences where the evaluations differed from predictions. Blue points show otherwise.

The evaluation of reconstruction coverage was calculated as a binary function, whether in the local neighbourhood any point was reconstructed or not. Figure 8 and Figure 9 present the example scenes with reconstruction coverage evaluation, predictions, and differences between them. Table 2 shows quantitative assessment of reconstruction coverage for different scenes types.

The results shown in Figure 8 and Figure 9 show a similar distribution of scores obtained from the prediction and actual data. Analysing the results obtained for a pair of cameras and for six cameras, one can notice the occurrence of differences (Figure 8c and Figure 9c) outside the main area of joint vision of the cameras. This is due to the detection and matching of groups of pixels located at the edges of spheres projected on images. Analysing their distribution, it should be stated that this does not affect the correctness of the proposed prediction model.

The values presented in Table 2 show that the relative error values of the prediction of the distribution of points used for the reconstruction of the reference sphere are between 2.8% to 4.1%. The analysis of the results shows that the value of the relative error increases as the number of cameras used for shape reconstruction increases. Only for the case of using two cameras, the value of this error is similar to the errors obtained for six and nine cameras.

#### 4.1.2. Density

**Figure 10:**
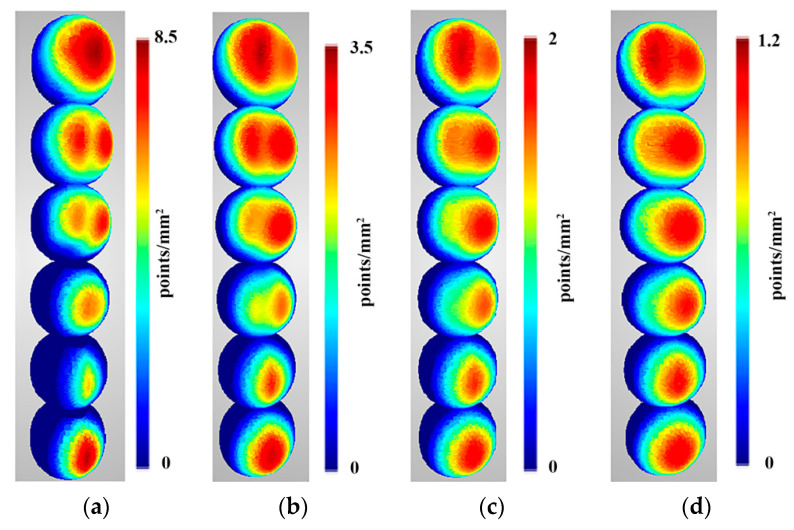
Density evaluation of the reconstructed sphere at the following distances: (**a**) 2 m, (**b**) 3 m, (**c**) 4 m, and (**d**) 5 m. Spheres in different rows were reconstructed from scenarios with different numbers of cameras. In order from bottom to top, these were as follows: two-, three-, four-, six-, nine-, and twelve-camera scenarios.

**Figure 11:**
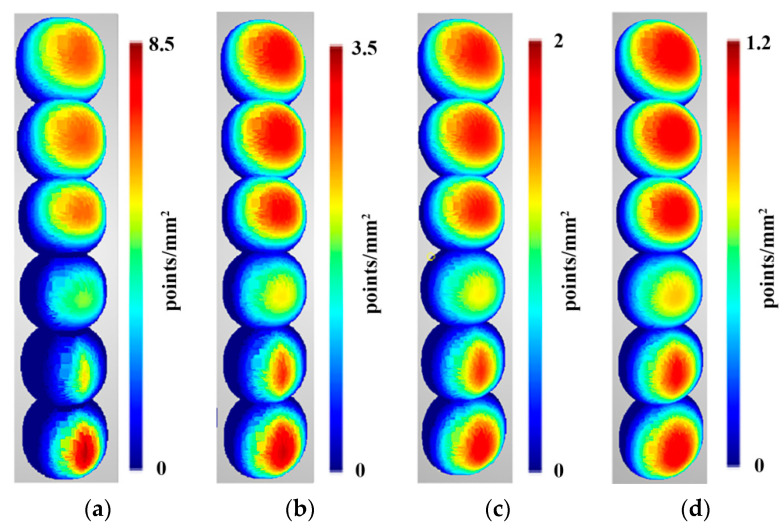
Density predictions of the reconstructed sphere at the following distances: (**a**) 2 m, (**b**) 3 m, (**c**) 4 m, and (**d**) 5 m. Spheres in different rows were reconstructed from scenarios with different numbers of cameras. In order from bottom to top, these were as follows: two-, three-, four-, six-, nine-, and twelve-camera scenarios.

**Figure 12:**
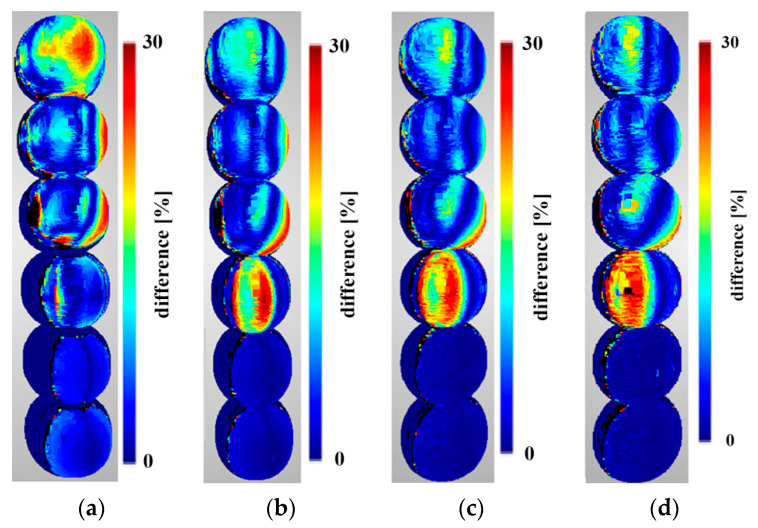
The relative difference between the predictions and evaluations of the density of the reconstructed sphere at the following distances: (**a**) 2 m, (**b**) 3 m, (**c**) 4 m, and (**d**) 5 m. Spheres in different rows were reconstructed from scenarios with different numbers of cameras. In order from bottom to top, these were as follows: two-, three-, four-, six-, nine-, and twelve-camera scenarios. Values higher than 30% are marked in black.

Density predictions were calculated according to functions described in Section 3.1. Below are presented density evaluations (Figure 10), density predictions (Figure 11) in the same scales per sphere to camera distance, and relative differences between them (Figure 12).

Analysing the distribution of point density values mapped using OpenMVS library functions (Figure 10), it can be observed that the density is not symmetric with respect to the centre of the reference sphere, although the cameras were distributed symmetrically. Moreover, it increases with the number of cameras regardless of the distance of the sphere from the cameras. Only for the case of two cameras, the highest density is obtained in the centre of the stereo pair view field, and the value is close to the density value obtained for 12 cameras. As in the case of a larger number of cameras, the density distribution does not change significantly with the distance but only for the number of points per mm^2^.

The obtained prediction for density distribution (Figure 11) is a symmetrical distribution with respect to the centre of the cameras’ field of view. Similar to the density distribution for the points detected using the OpenMVS library, it can be observed that the density of the points increases with increasing number of cameras, starting from three. The same dependence of the distribution on the number of points can be observed for two cameras as in the case with real values.

When analysing the results shown in Figure 12, no significant differences in density variation can be seen between the point clouds reconstructed using two and three cameras. Only differences at the edge of the reconstructed area can be observed. For the closest distance (2 m), the most significant variation in density can be seen for point clouds reconstructed from 12 cameras, while for the other cases (3, 4, and 5 m), this for the greatest variation can be observed with four cameras. A similar value of the variance of the differences in densities was obtained for point clouds mapped from six and nine cameras for all distances between the reference sphere and the cameras. In Figure 12, the areas for which the value of difference exceeds 30% are shown in black, and will be treated as “outliers” in future investigations.

Table 3 presents a quantitative assessment of the differences between prediction and evaluation for all points in the dataset with the outliers removed.

The results presented in Table 3 show that the average percentage value for differences in density for point clouds obtained are similar for scenarios with two and three cameras, and are below 3%. Scenes containing six, nine and twelve cameras have an average difference between 8% and 11%. The highest average difference value was obtained for the scenario with four cameras, although this did not exceed 16%. The values of RMS of differences are in the range from 0.019% to 0.118%, and are characterised by a narrow dispersion. Analysis of the median and standard deviation confirms the relationship observed for the mean and RMSE, confirming the validity of the adopted point cloud density prediction model.

#### 4.1.3. Accuracy

**Figure 13:**
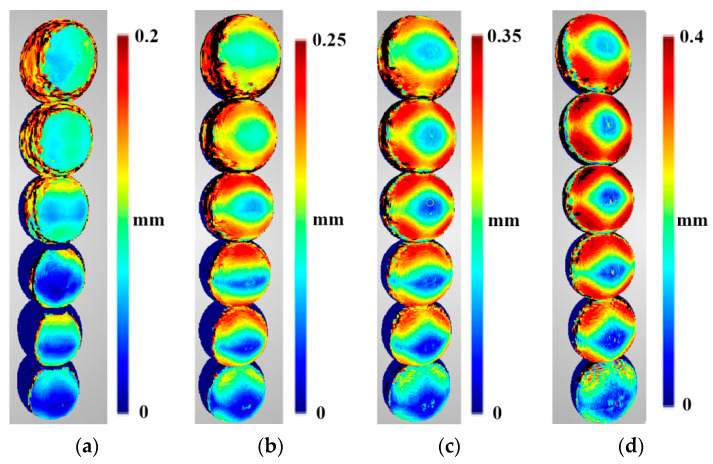
Evaluation of the accuracy of the reconstructed sphere at the following distances: (**a**) 2 m, (**b**) 3 m, (**c**) 4 m, and (**d**) 5 m. Spheres in different rows were reconstructed from scenarios with different numbers of cameras. In order from bottom to top, these were as follows: two-, three-, four-, six-, nine-, and twelve-camera scenarios. Values higher than 0.2 mm for 2 m, 0.25 mm for 3 m, 0.35 mm for 4 m and 0.4 mm for 5 m, respectively, are marked in black.

**Figure 14:**
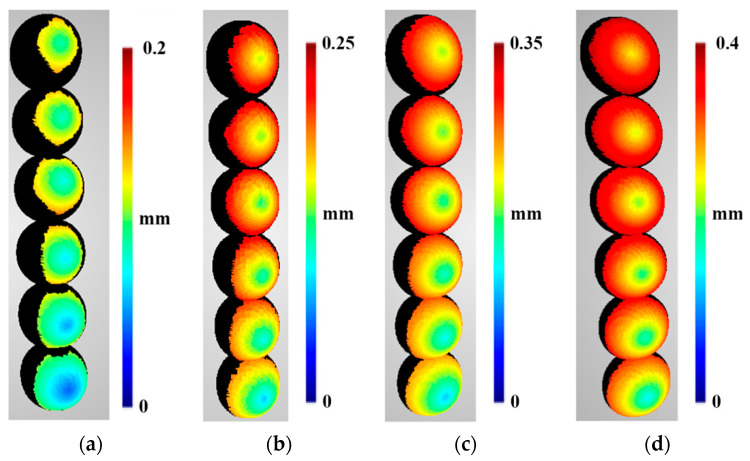
Predicted accuracy of the reconstructed sphere at the following distances: (**a**) 2 m, (**b**) 3 m, (**c**) 4 m, and (**d**) 5 m. Spheres in different rows were reconstructed from scenarios with different numbers of cameras. In order from bottom to top, these were as follows: two-, three-, four-, six-, nine-, and twelve-camera scenarios. Values that are higher 0.2 mm for 2 m, 0.25 mm for 3 m, 0.35 mm for 4 m and 0.4 mm for 5 m, respectively, are marked in black.

**Figure 15:**
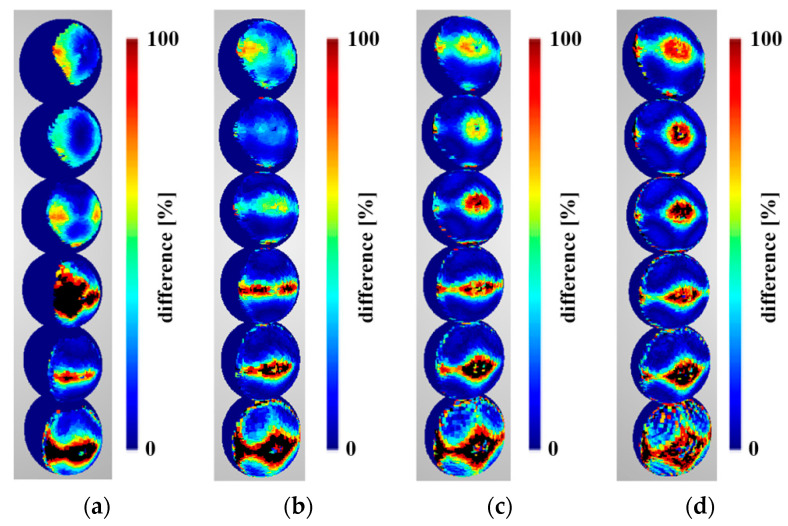
The relative difference between the accuracy predictions and evaluations of the reconstructed sphere at the following distances: (**a**) 2 m, (**b**) 3 m, (**c**) 4 m, and (**d**) 5 m. Spheres in different rows were reconstructed from scenarios with different numbers of cameras. In order from bottom to top, these were as follows: two-, three-, four-, six-, nine-, and twelve-camera scenarios. Values higher than 100% are marked as black.

Accuracy predictions were calculated according to the functions described in Section 3.1. The pixel error in Equation (5) was determined during the optimisation process to be 0.1365 px. Below, the accuracy evaluations (Figure 13) and accuracy predictions (Figure 14) are presented using the same scale for sphere-to-camera distance, along with the relative differences between them (Figure 15).

The results in Figure 13 show that as the number of cameras increases, the error in the shape of the reference sphere increases to 0.2 mm for distances of 2 m, to 0.25 mm for 3 m, to 0.35 for 4 m, and to 0.4 mm for 4 m, respectively. As the distance between the imaged object and the cameras increases, the symmetry in the four directions is noticeable, with the smallest error being in the central part, and increasing linearly toward the edge. For shorter distances and fewer cameras (two, three, and four), there is symmetry in the error distribution with respect to the centre in two directions (up and down). Similar to the density evaluation results, outliers in the dataset are marked as black boxes in Figure 13. The erroneous determination of points in these regions was caused by the matching algorithm on the edges of the circle of the projected spheres in the images.

Moreover, it can be observed that at the edges of the accuracy prediction maps, extreme values are present. This is because for MVS reconstructions, cameras observing a given point from a high angle to the surface normal vector bias the accuracy significantly. It can be clearly seen in Figure 13a for the twelve-camera scenario for the sphere at a distance of 2 m that the accuracy increases rapidly at the edge of the points visible to all cameras. Therefore, because this phenomenon needs to be characterised by a different model than that proposed in this paper, only points visible to all cameras in the given scene are used.

It can be noticed that a similar distribution is present in the accuracy predictions as that present in the evaluations (Figure 14). The accuracy decreases (accuracy values in millimetres increase) with: the camera-to-object distance; the number of cameras; and angle of the normal vector to the camera observation direction. Although the predictions are much more symmetrical than the evaluations, the most important feature for view planning is the rough similarity in the obtained distributions and values.

Figure 15 presents the relative differences between the predictions and the evaluations, and Table 4 shows the quantity assessment for all points in the dataset without outliers. The results shown in Figure 15 show that the most significant differences between the accuracy prediction and the evaluation were when using two cameras at all distances. It should be emphasised that the predicted accuracy was lower than that obtained on the basis of the measured data.

The results presented in Table 4 show that the mean percentage values of differences in the determination of accuracy for point clouds acquired using three to nine cameras are roughly equal at approximately 21%. The highest average value of differences was obtained for two cameras at 50.84%. The RMS values range from 0.29% to 0.44% and are characterised by a small dispersion. Analysis of the median and standard deviation confirms the relationship observed for the mean and RMSE, confirming the validity of the adopted point cloud density prediction model.

### 4.2. 360-Degree Scene Camera Setup Generation

To choose the best camera network, as described in Section 3.1.1, 10,000 random camera setups were generated. The measurement volume was defined as a 2.6 × 4.0 × 2.0 m cuboid filled with 216 points arranged in a grid. For each point, 100 directions were generated, evenly distributed on the sphere using the Fibonacci algorithm [51]. Predictions were calculated for each point and each direction. The statistical results of the prediction obtained for the generated camera setups varied within the following ranges:

- Reconstructible direction ratio for all points: (46.5%, 93.6%);
- Average density: (0.32, 0.81);
- Average accuracy: (0.21, 0.72 mm).

**Figure 16:**
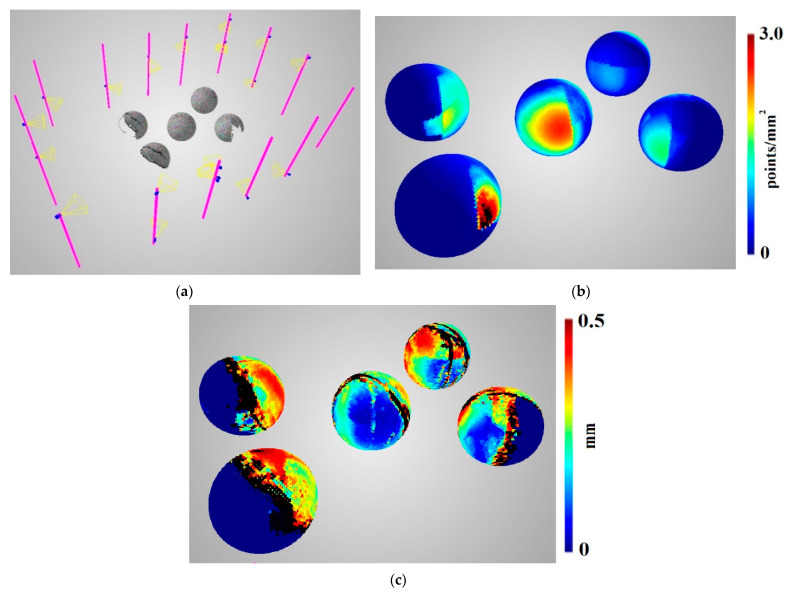
Average 360-degree scene with (**a**) 20-camera distribution and 5 reconstructed spheres in the measurement volume with (**b**) density and (**c**) accuracy evaluations. Values that are higher than 3.0 in (**b**) and 0.5 in (**c**) are marked in black.

**Figure 17:**
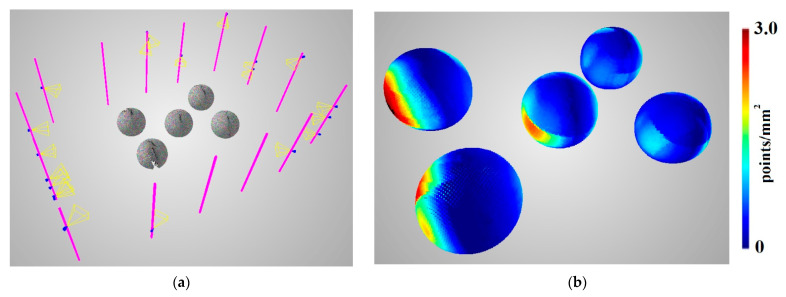

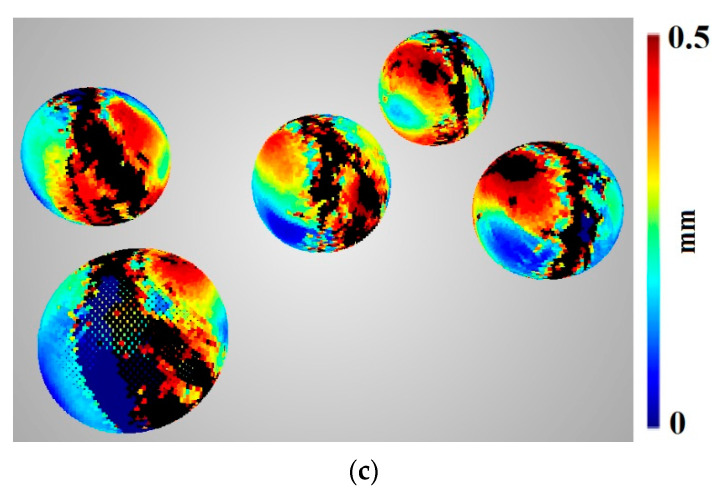
Quasi-optimal 360-degree scene with (**a**) 20-camera distribution and 5 reconstructed spheres within the measurement volume with (**b**) density and (**c**) accuracy evaluations. Values that are higher than 3.0 in (**b**) and 0.5 in (**c**) are marked in black.

For the purposes of this paper, two camera setups were chosen for comprehensive analysis. The first one chosen was selected to be approximately in the middle of the statistical range of the presented predictions, and was called the average setup (Figure 16). The second one was the quasi-optimal setup (Figure 17), selected by the operator based on the obtained prediction statistics. Choosing the best setup was difficult, because multiple statistics were generated for each setup. There is no straightforward cost function that can be sued to compare two setups with reference to a single scalar value. In the presented case, the priority was to obtain the best possible coverage with reasonable density and accuracy. Below, the average and quasi-optimal setups are shown, individually rendering and reconstructing the same five spheres inside the measurement volume.

Analysing Figure 16, it can be seen that the average camera setup results in partial reconstruction of the objects inside the measurement volume. The spheres visible in Figure 16 on the left side are reconstructed to an extent of less than 50%, and only the central one is reconstructed to an extent of 89.2%.

Compared to the average setup, the quasi-optimal camera setup presented in Figure 17 resulted in a much more complete reconstruction and higher density. A quantitative comparison of these two scenes is presented in Table 5. The quasi-optimal scene covers 92.3% of the reconstructed spheres’ surfaces, although the spheres’ bottom surfaces are only 0.5 m above the column’s ground level (meaning that for the bottom parts of the spheres, the observation direction of the cameras is close to 90 degrees, and thus, reconstruction is almost impossible). Similarly, the quasi-optimal camera setup reconstruction’s density is much better than that of the average setup. The median density value is almost two times higher for the quasi-optimal setup than for the average one. Only the accuracy is better for the average setup; however, this could be because it has less coverage, and non-reconstructed points were not included when calculating the average and median accuracy.

Finally, the prediction functions need to be validated for the two camera setups described above. Quantitative assessments of coverage, density, and accuracy predictions with are presented respect to their evaluations in Table 6. It can be seen that coverage predictions correspond well with their evaluations. The predictions of density and accuracy are less equivalent; however, the distribution is similar, as shown in Appendix A in Table A1 and Table A2, which is crucial for image network planning.

### 4.3. 360-Degree Real Camera Setup

**Figure 18:**
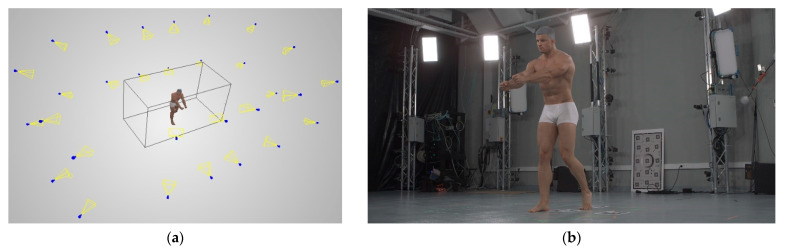
The measurement system used for 3D reconstruction of real data: (**a**) camera setup with measurement region (black cuboid) and example human body reconstruction, (**b**) image captured by an example camera using the measurement system.

The proposed prediction functions were tested on real data, and images were acquired using 30 RGB Flir Oryx 10Gige cameras with a resolution of 4096 × 2196 (4K). A single person was chosen as the measured object. The subject’s full body was measured using a 360-degree setup. The camera setup was used to perform measurements within the measurement region, and an example reconstruction is presented in Figure 18. For 3D shape reconstruction, OpenMVS v1.1.1 was used.

**Figure 19:**
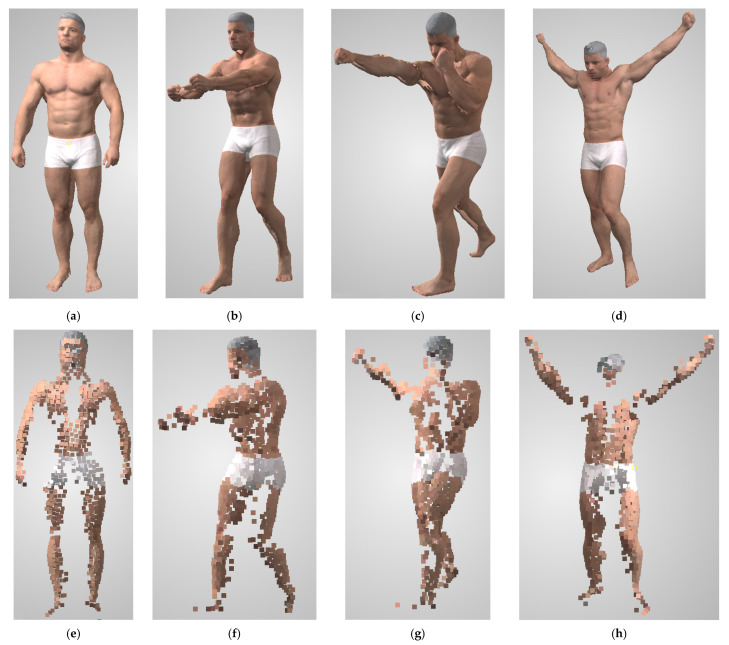
3D reconstructions of the human in different poses: (**a**) pose 1, (**b**) pose 2, (**c**) pose 3, (**d**) pose 4, with corresponding unoccluded sparse point clouds (**e**–**h**).

To evaluate the prediction functions, the 3D shape reconstructed from a human body in four different poses and positions was calculated (Figure 19a–d). Analogous to the synthetic datasets presented in this paper, from reconstructed dense point clouds, sparse point clouds with points separated by approximately 30 mm were generated. Normal vectors were calculated with local plane fitting on dense clouds. Because prediction functions do not consider occlusions, occluded points were removed from the sparse point clouds. Unoccluded sparse point clouds corresponding to 3D reconstructions are presented in Figure 19e–h.

**Figure 20:**
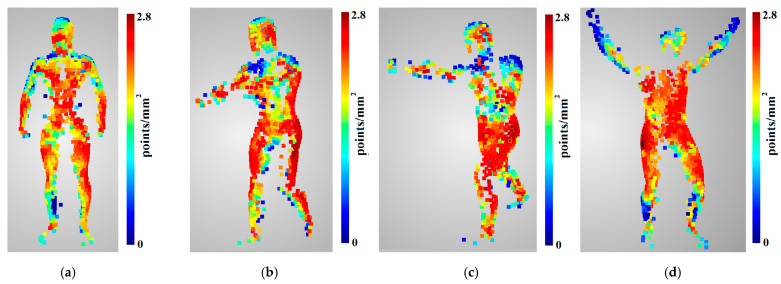
Sparse point clouds of the subject in different poses: (**a**) pose 1, (**b**) pose 2, (**c**) pose 3, (**d**) pose 4, with density evaluations presented as colormaps.

The predictions and evaluations were calculated for each point in the unoccluded sparse point clouds (Figure 19e–h). The lack of ground truth geometry from real data prevented the evaluation of the accuracy; thus, predictions and evaluations were calculated only for coverage and density. Because the points in the sparse point cloud were generated from the dense point cloud, the evaluated coverage was equal to 1 in all of them. However, for each of them, in all poses, the predicted coverage was also equal to 1. Thus, coverage predictions were 100% correct. The evaluated densities are presented in Figure 20, the statistics in Table 7, and the prediction-to-evaluation difference statistics in Table 8.

Analysing the densities of the 3D reconstructions of the four poses presented in Figure 20, it can be observed that the lowest densities were located in the upper parts of the body with normal vectors directed upwards, and in the lower parts of the body. This is because the cameras were placed around the scene, with no cameras observing the subject from the bottom or from the top. The highest densities were obtained in the middle of the 3D model.

The results presented in Table 7 show that the average and median densities for all poses are similar. The average density values are in the range from 1.725$\frac{\mathrm{points}}{{\mathrm{mm}}^{2}}$ for pose 1 to 1.909$\frac{\mathrm{points}}{{\mathrm{mm}}^{2}}$ for pose 2. The lowest median value was similar to the average value obtained for pose 1; however, the highest median value was obtained for pose 4. The statistics presented in Table 8 show that the mean percentage values of differences in density determination for the point clouds are in the range between 33.8% and 45%. The highest average difference value was obtained for pose 4. The RMS values are in range from 1.39% to 1.71%, and are one order of magnitude higher than those obtained for the synthetic data presented in Section 4.2; however, they are still characterised by a narrow dispersion. Analysis of the median and standard deviation confirms the relationship observed for the mean and RMSE, confirming the validity of the adopted point cloud density prediction model.

### 4.4. Summary

The results section presented the process of optimising the coverage, density and accuracy prediction functions using synthetic spheres and the validation of this method using 360-degree scenes, both synthetic and real. Ten thousand camera setups were randomly generated. The predictions and their statistics were calculated for discretised measurement volumes in multiple directions for each set. Two camera setups were selected for validation: the first was the average one in terms of the prediction statistics, and the second was the quasi-optimal one chosen by the operator. Images of five synthetic spheres were rendered, and their 3D reconstructions were calculated. Evaluation of the reconstructions proved that the selected quasi-optimal camera setup resulted in much better reconstruction coverage and density. Finally, the prediction functions were assessed on the basis of real data of a single person in different poses and positions in real prepared camera setups. The predicted coverage was fully consistent with the evaluated coverage, and prediction-to-evaluation differences in average density did not exceed 45.0%.

## 5. Discussion

The proposed automatic image network design algorithm is based on three main steps: (1) determine the input parameters, including volume and size, camera type, and occlusion areas; (2) define the main algorithm for camera position determination, discretising the measurement volume with a regular point grid, with the best camera setup being selected using a Monte Carlo-based algorithm, and (3) determine the final camera position on the basis of the prediction functions of coverage, density and accuracy, along with the extended geometrical analysis. The proposed algorithm uses 3D shape reconstruction methods from the open-source OpenMVS library. We opted for only one MVS algorithm due to the fact that the OpenMVS library is an open-source solution that can be used and modified according to the license terms. Because the OpenMVS library is open source, it is possible to analyse the assumptions and performance of the various algorithms used for 3D reconstruction. The OpenMVS project is constantly under development. In the official repository, it is possible to follow all the changes and, if necessary, modify the assumptions of the proposed approach in the article.

Conventional image network design algorithms (for both static and dynamic scenes) assume a priori knowledge about the object shape (for static scenes) or the approximate position of a moving object (for dynamic scenes). By applying volumetric scene discretisation using the proposed algorithm, it is possible to simulate the position of both static and moving objects. This extends the camera functionality, as optimisation is performed for multiple accuracies, GSD sizes, and densities achievable from all cameras for different acquisition distances. By defining multiple regions with different densities and accuracies, it is possible to instantly optimise camera position. The commonly used approaches assume that all cameras are looking at the centre of the measurement area. Our method enables an increased measurement area and accuracy through the use of the optimal number of cameras.

It should be noted that the tests were carried out on both ideal synthetic data and real data. In the proposed approach for determining the optimal number of cameras necessary reconstructing an object in motion within the defined measurement volume, certain simplifications were adopted concerning, among other things, the types of cameras used, the impact of lighting, and the selection of the texture of the object to be reconstructed. An ideal pinhole camera model without the influence of radial and tangential distortion error was assumed, without depth-of-field determination.

To assess the correctness of the adopted concept of dynamic (4D) Multi-View Stereo camera system network design, camera parameters corresponding to those of the Flir Oryx 10Gige camera with 4k resolution were used. This sensor enables acquisition at 68 frames per second. The use of higher-resolution sensors might improve the accuracy of 3D point reconstruction. It was decided to use a single type of lens with a focal length of 16 mm. It should be noted that the use of different lenses significantly affects the field of view of individual cameras as well as their position in space, and, if the difference in magnification between the pair of images is no greater than 2.4, it is possible to use a stereo pair to perform 3D shape reconstruction.

To evaluate the degree of influence of texture on the accuracy and completeness of the shape representation using the algorithms implemented in the OpenMVS library, five types of characteristic patterns were used, namely RGB noise (random colours), colour triangles, stained glass, random colour line segments, and random black points. The analysis shows (Table 1) that the best results, i.e., the lowest RMSE value of the reference sphere and the largest number of points, were obtained for RGB noise (random colours). This approach allowed us to eliminate the influence of texture quality on the accuracy of the shape representation and to explicitly analyse the quality of the proposed prediction functions on the basis of the accuracy, density, and visibility of points from as many cameras as possible. The pixel error value (Equation (5)) would be optimised again when considering a different texture type. It should be emphasised that the aim of this article was not to analyse the influence of different textures on the quality of shape reproduction using the functions of the OpenMVS library.

**Figure 21:**
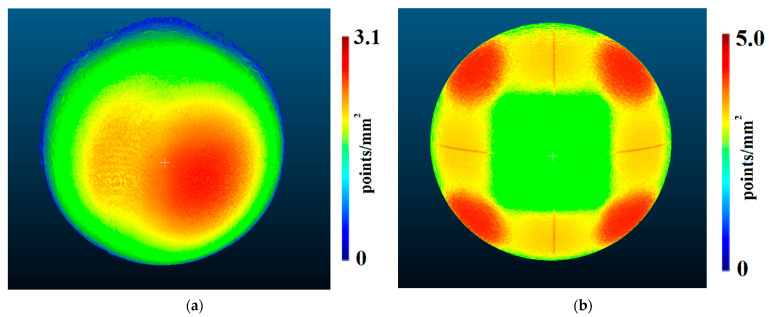
The differences in the point distribution obtained by the (**a**) OpenMVS—Patch-Based Stereo Method; and (**b**) Agisoft Metashape—Semi-Global Matching from the same synthetic images.

Analysing the results obtained for density of points per mm^2^, the asymmetry of the distribution can be observed. This is due to the algorithm used to detect mismatched characteristic areas on stereo pairs (Patch-Based Stereo Method; Normalized Cross-Correlation) and the Depth-Map Refinement and Merging method adopted (Figure 10). The proposed algorithm cannot be used explicitly with the Semi-Global Matching algorithm, which is implemented, e.g., in the commercial software Agisoft Metashape, because when comparing the density results obtained for six cameras, a significant difference in the distribution of points can be observed (Figure 21).

It should be stated that the reconstruction’s completeness is crucial for 3D/4D measurements. Once full object coverage has been obtained, further examination of density and accuracy should proceed. Calculating predictions in multiple directions for each point is a similar approach to that presented by Rahimian et al. [36]. This is called the dynamic occlusions model; however, it uses only the Boolean function, whether the point from a given direction will be reconstructed or not. Our approach improves on this and allows us to also calculate predictions for density and accuracy at a given point from a given direction.

The proposed formula achieved a maximum mean error of 0.5 mm between predictions and evaluations in the 2.6 × 4.0 × 2.0 m measurement volume scene. For accuracy prediction, unlike in [36], we used observation direction and distance between given 3D points and the cameras, and not only the angle between the camera pair. Using supervised selection (Figure 17) of the best camera positions based on the calculated statistical predictions, it was possible to cover 92.3% of the reconstructed sphere surfaces, although the sphere’s bottom surface was only 0.5 m above the column’s ground level (meaning that reconstruction from the bottom was impossible). Tests on real data of the full the body of a single person resulted in confirmation of coverage predictions and the obtaining of average differences between predictions and evaluations of density no greater than 45.0%. The error in the predictions of real data were higher than that for synthetic data. This was due to several reasons: (1) real data are encumbered by thermal noise, (2) both interior and exterior orientation camera parameters were calculated with errors, (3) the illumination and camera exposure do not allow the use of the full bit depth, and (4) the texture of the measured subject was not optimal for SfM/MVS algorithms. However, despite higher rates of prediction-to-evaluation error than on synthetic data, the proposed prediction functions are sufficient for the proposed image network design algorithm.

We claim that our approach is more precise; however, it requires specific point coordinates for calculation. Moreover, we estimated error not as a constant value in the given range in the camera’s axis angles, but as a continuous function that considers the camera’s relative transformation and the measured point’s coordinate and normal direction.

## 6. Conclusions

Image network design is a critical factor in image-based 4D shape reconstruction and data processing (especially in the application of combined SfM/MVS methods). The value of the proper spatial distribution increases with the amount of data captured during 4D scene reconstruction. This is crucial for time-varying scene acquisition that allows real-time object tracking based on the whole shape of the objects under investigation, rather than a point representation. Good scene planning can significantly reduce image processing time (using Multi-View Stereo algorithms) and camera count while maintaining the accuracy and completeness of shape representation of the moving from all detectors.

The presented image network design method does not assume approximate knowledge of the shape to be detected or the approximate position of the object in motion, but only the measured volume. Because the approach allows the discretisation of surfaces of any shape, it is possible to guarantee the visibility of any “movable” object from all possible directions. The proposed approach based on prediction functions taking into account accuracy and resolution in all possible positions of a moving object (dynamic scenes) in volumetric measurements is a unique approach that can be implemented in any study area. It should be emphasised that this is the first method enabling the estimation of accuracy for volumetric measurements.

The proposed method was tested on a man moving within a 2.6 m by 4.0 m by 2.0 m volume. Thanks to the algorithms used and the approach based on a user-defined processing area, this approach can also be used to improve on the rendering and subsequent clinical application presented, for example, in Alhammadi [52].

In further research related to the development of dynamic image network design algorithms, it is planned to consider parameters such as radial and tangential distortions and select the f-number for a given camera to calculate its depth.

## Figures and Tables

**Table 1:** Comparison of reconstructions of the same sphere in a single scenario with different textures with number of points and reconstruction accuracy.

Texture Type	Number of Points	The RMSE of Deviations from 500 mm Sphere [mm]
RGB noise (random colours)	11,497,207	0.26
Colour triangles	7,307,546	0.52
Stained glass	9,722,841	0.67
Random colour line segments	10,683,159	0.33
Random black points	11,708,933	0.33

**Table 2:** Invalid reconstruction coverage predictions ratio for different scene types.

Number of Cameras	Error Predictions [%]
2-camera	3.75
3-camera	2.86
4-camera	2.79
6-camera	3.9
9-camera	3.8
12-camera	4.1

**Table 3:** Statistics for the differences between predicted and evaluated density under different scenarios with outliers removed.

Scene Type	Average Difference (%)	RMS Difference (%)	Median Difference (%)	Standard Deviation (%)
2-camera	2.49	0.019	1.97	1.84
3-camera	2.83	0.039	2.14	2.26
4-camera	15.97	0.019	15.05	10.68
6-camera	10.72	0.118	9.19	7.19
9-camera	8.79	0.089	7.28	5.94
12-camera	10.73	0.099	9.81	5.61

**Table 4:** Statistics for differences between predicted and evaluated accuracy under different scenarios with outliers removed.

Scene Type	Average Difference (%)	RMSE (%)	Median Difference (%)	Standard Deviation (%)
2-camera	50.84	0.35	44.79	26.29
3-camera	21.37	0.29	15.83	16.79
4-camera	21.95	0.32	15.54	17.76
6-camera	20.93	0.29	15.46	15.78
9-camera	20.05	0.29	16.48	13.11
12-camera	28.89	0.44	25.85	17.41

**Table 5:** Statistics of coverage, density, and accuracy evaluation for different setups without outliers.

Value	Average Setup	Quasi-Optimal Setup
Coverage	68.2%	92.3%
Average density	$0.53\;\;\frac{\mathrm{points}}{{\mathrm{mm}}^{2}}$	$0.78\;\frac{\mathrm{points}}{{\mathrm{mm}}^{2}}$
Median density	$0.35\;\;\frac{\mathrm{points}}{{\mathrm{mm}}^{2}}$	$0.67\;\frac{\mathrm{points}}{{\mathrm{mm}}^{2}}$
Average accuracy	0.29 mm	0.42 mm
Median accuracy	0.19 mm	0.28 mm

**Table 6:** Statistics for the difference between prediction and evaluation for different setups with outliers removed.

Value	Statistics Type	Average Setup (%)	Quasi-Optimal Setup (%)
Coverage	Error prediction	5.38	3.36
Density	Average difference	32.0	23.17
	RMSE	0.38	0.21
	Median difference	25.53	21.42
	Standard deviation	26.73	12.07
Accuracy	Average difference	36.85	34.00
	RMSE	0.41	0.32
	Median difference	28.9	28.67
	Standard deviation	25.38	22.10

**Table 7:** Density evaluation statistics for different poses of a human.

Value	Pose 1	Pose 2	Pose 3	Pose 4
Average density	$1.725\frac{\mathrm{points}}{{\mathrm{mm}}^{2}}$	$1.909\frac{\mathrm{points}}{{\mathrm{mm}}^{2}}$	$1.829\frac{\mathrm{points}}{{\mathrm{mm}}^{2}}$	$1.896\frac{\mathrm{points}}{{\mathrm{mm}}^{2}}$
Median density	$1.802\frac{\mathrm{points}}{{\mathrm{mm}}^{2}}$	$1.971\frac{\mathrm{points}}{{\mathrm{mm}}^{2}}$	$1.909\frac{\mathrm{points}}{{\mathrm{mm}}^{2}}$	$1.987\frac{\mathrm{points}}{{\mathrm{mm}}^{2}}$

**Table 8:** Statistics for the differences between predicted and evaluated density for different human poses with outliers removed.

Value	Statistics Type	Pose 1 (%)	Pose 2 (%)	Pose 3 (%)	Pose 4 (%)
Density	Average difference	39.8	44.2	33.8	45.0
	RMSE	1.43	1.71	1.39	1.63
	Median difference	40.9	45.7	35.6	46.4
	Standard deviation	9.97	9.74	10.69	8.09

## Appendix A

**Table A1:** Comparison of predictions and evaluations of an average 360-degree scene from Figure 16: (a) reconstruction’s coverage (red points represent covered areas for evaluations and predictions, and for relative difference, invalid predictions), (b) density, and (c) accuracy.

	Evaluations	Predictions	Relative Difference
(a)	****		
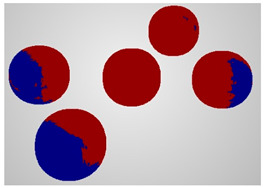
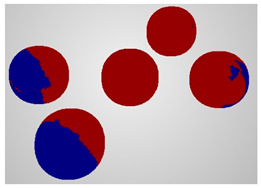
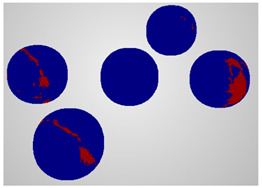
(b)	****		
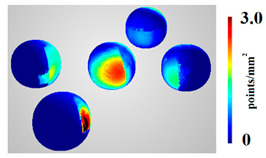
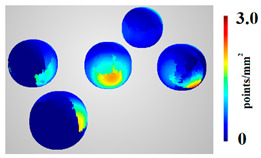
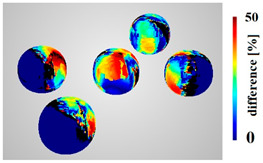
(c)	****		
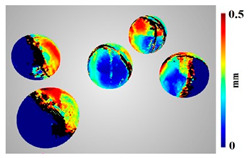
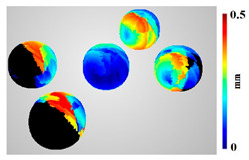
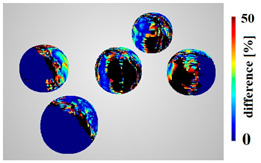

**Table A2:** Comparison of predictions and evaluations of a quasi-optimal 360-degree scene from Figure 17: (a) reconstruction’s coverage (red points represent covered areas for evaluations and predictions, and for relative difference, invalid predictions), (b) density, and (c) accuracy.

	Evaluations	Predictions	Relative Difference
(a)	****		
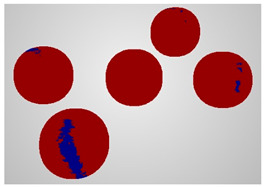
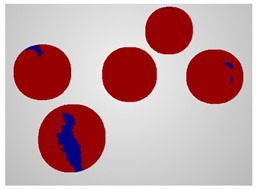
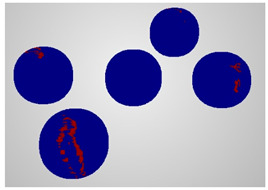
(b)	****		
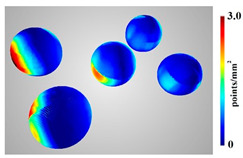
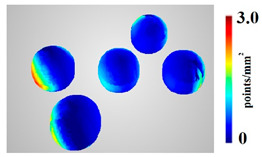
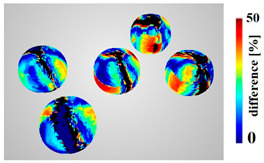
(c)	****		
